# Correlation between COVID-19 case fatality rate and percentage of BCG vaccination: is it true the vaccine is protective?

**DOI:** 10.1186/s43168-020-00022-1

**Published:** 2020-09-09

**Authors:** Aliae A. R. Mohamed Hussein, Marwa Rashad Salem, Samar Salman, A F Abdulrahim, Nasrallah A. Al Massry, Mahmoud Saad, Nesrine Ben Hadj Dahman, Ahmed Negida

**Affiliations:** 1 Assiut University Hospitals, Assiut, 71515 Egypt; 2 Epidemiology and Public Health, Cairo University, Giza, Egypt; 3 Dermatology and Venerology, Faculty of Medicine, Tanta University, Tanta, 30255 Egypt; 4 Assiut University, Assiut, 71515 Egypt; 5 Faculty of Medicine, Al-Quds University- Palestine, Al-Azhar branch- Gaza, Gaza, State of Palestine; 6 Faculty of Medicine of Tunis, Tunis, Tunisia; 7 Neurosurgery Department, Zagazig University Hospitals, Zagazig University, Sharkia, Egypt; 8 School of Pharmacy and Biomedical Sciences, University of Portsmouth, Portsmouth, UK

**Keywords:** Coronavirus disease, SARS-Cov-2, COVID-19, BCG vaccination, Tuberculosis vaccine

## Abstract

**Background:**

The observations of some recent epidemiological studies offer hope for a reduced impact of COVID-19 for countries which practice universal BCG vaccination policy.

**Main body:**

This report provides a correlation between the case fatality rates of COVID-19 and the percentage of BCG vaccination coverage in 183 most affected countries. The main objective of this observational ecologic report is to evaluate possible effects of the previous BCG vaccination in different populations and the epidemic outcomes specially the rates of severe/critical cases and case fatalities. The analysis is preliminary since it is based on constantly rolling data while the COVID-19 pandemic is still unfolding.

**Conclusion:**

Our findings seem to support the fact that an older BCG vaccine may have a protective role in avoiding severe/critical SARS-CoV2 pneumonia and relatively decrease its fatalities.

## Background

Several reports showed that exaggerated immune response of COVID-19 contributes to severe disease [1]. On May 19th, 2020, the number of confirmed cases is 4,956,733 with total deaths 323,095, with a fatality rate of 0.22 per million. All over the world, twenty-six of the 179 countries had unknown status regarding BCG vaccination, while 132 have current BCG vaccination programs and 21 have no national BCG program.

As the BCG vaccine is already approved for human use, it is known to boost the immune system and modulate the exaggerated immunity response against tuberculosis [2]. It provides protection against a range of infections, not simply the mycobacterium for which the vaccine was originally developed [3] but also a range of conditions, including respiratory infections (bacterial and viral), neonatal sepsis, and fevers [4], and geriatric acute upper respiratory tract infections [5].

It is hypothesized that, until a specific vaccine is developed, SARS-CoV-2 vulnerable populations could be immunized with BCG vaccines. Such a strategy would also be suitable for frontline health personnel [6].

*The aim of this perspective* is to correlate between the COVID-19 case fatality rates, serious/critical case percentage, and percentage of national BCG vaccine coverage.

In *methodology*, to evaluate the effect of BCG vaccine, we included the BCG coverage percentage in 183 countries (countries which have never included BCG vaccine in their national immunization program (NIP), countries which had included the vaccine in the past but do not do so currently, and countries who currently have BCG vaccine included in their NIP) and the COVID-19 infection rate, mortality levels, prevalence of serious/critical cases, and case fatality rates (Table 1). Pearson correlation was done to test the association of these variables.

**Table 1:** Correlations of BCG coverage percentage in 183 countries (countries that follow a national BCG immunization program and those that did not have or have ceased their national BCG vaccination programs) and the number of cases/1000 population and number of serious and critical cases/1000 and deaths/1000 population and case fatality rates (in 121 countries)

Variable		BCG coverage%	Cases/1000 population^**1**^	Serious cases/1000 population^**2,3**^	Deaths/1000 population^**4**^	CFR^**5**^
**BCG coverage**	Pearson correlation	1	− 0.592-^**^	− 0.256-^**^	− 0.608-^**^	− 0.388^**^
	Sig. (2-tailed)		< 0.001	0.001	< 0.001	< 0.001
	*N*	180	180	180	180	121
**Death rate**	Pearson correlation	− 0.608-^**^	1	0.321^**^	1	1
	Sig. (2-tailed)	< 0.001	–	< 0.001	1	–
	*N*	180	183	183	183	121

Coronavirus related statistics were based on data obtained from https://www.worldometers.info/coronavirus/ (according to the latest update on May 2nd, 2020, 20:44 GMT). BCG related statistics were based on Global Health Observatory data repository, BCG Immunization coverage estimates by WHO region, 2018. Doi:10.1371*/*journal*.*pmed*.*1001012*.*g002

^1^Statistically significant negative moderate correlation (*R* = − 0.592) between BCG coverage and number of cases/1000 (incidence rate) of COVID-19 among the studied countries, *P* < 0.001

^2^Statistically significant negative correlation (*R* = − 0.25) between BCG coverage and number of serious cases/1000 (incidence rate of COVID-19 serious cases) among the studied countries, *P* = 0.001

^3^Statistically significant positive correlation (*R* = 0.32) between death rate and incidence rate of COVID-19 serious cases among the studied countries at *P* value < 0.001

^4^Statistically significant negative moderate correlation (*R* = − 0.6) between BCG coverage and death rates at *P* value < 0.001

^5^Statistically significant negative weak correlation (*R* = 0.38) between BCG vaccination coverage and case fatality rate of COVID-19 among the studied countries at **=P value < 0.001

**Fig. 1:**
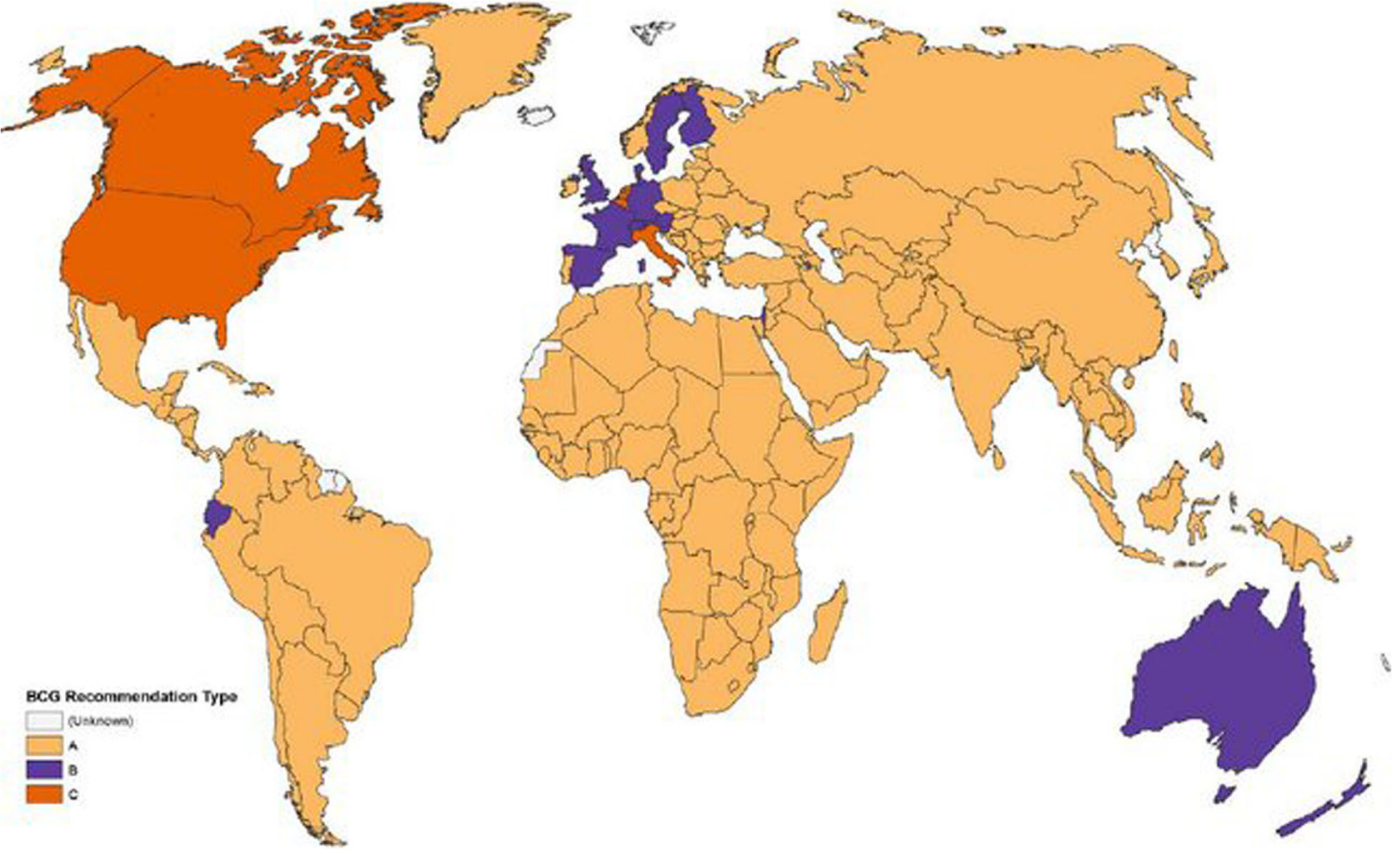
Map displaying BCG vaccination policy by country. **a** The country currently has universal BCG vaccination program. **b** The country used to recommend BCG vaccination for everyone but currently does not. **c** The country never had universal BCG vaccination programs. doi:10.1371/journal.pmed.1001012.g002

**Fig. 2:**
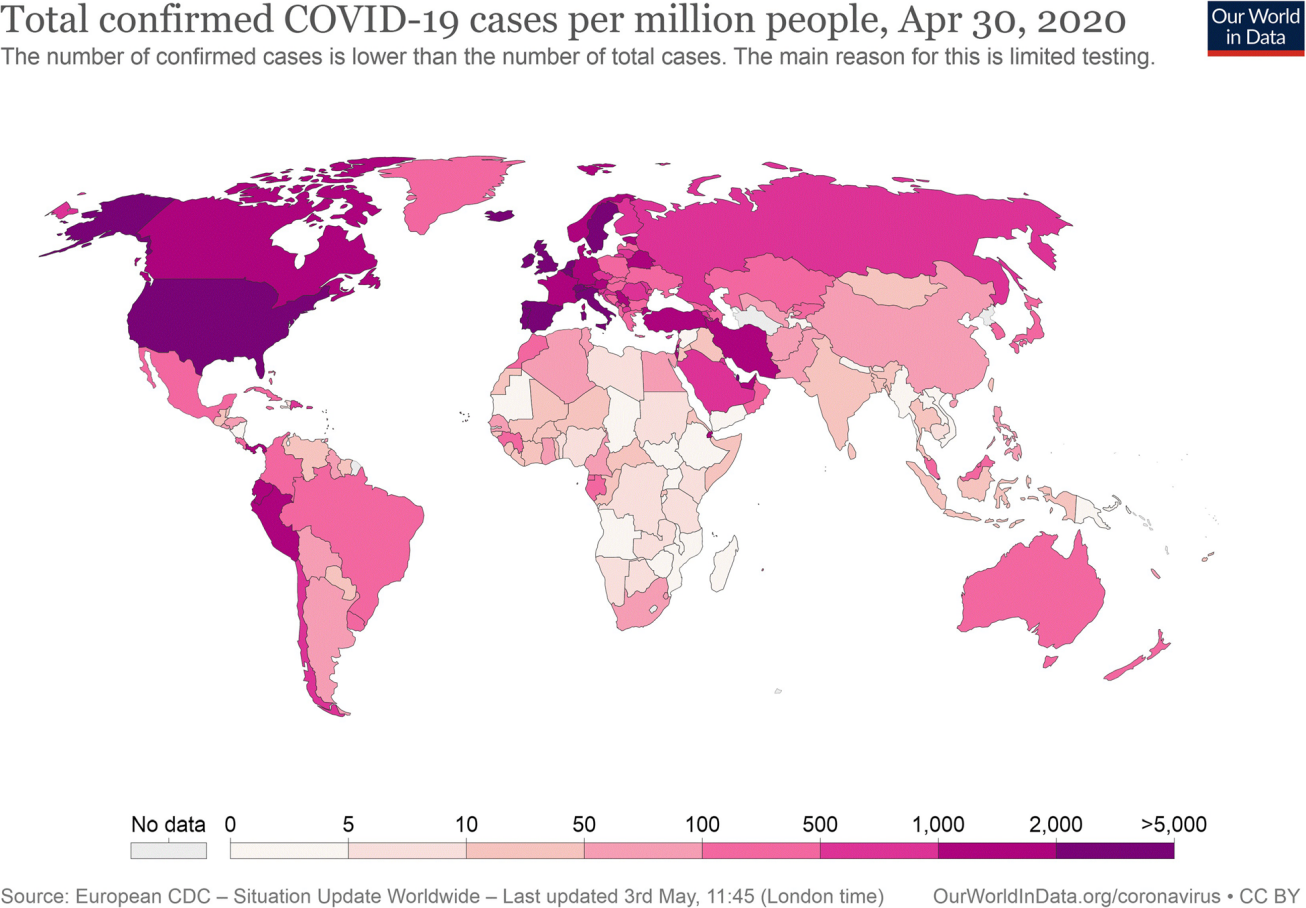
Map of the COVID-19 verified number of infected per capita *as of 2nd May 2020*. Since this is a rapidly evolving situation, new cases may not be immediately represented visually. Refer to the primary article 2019–20 coronavirus pandemic or the World Health Organization’s situation reports for most recent reported case information. Every country bigger than 3 million km^2^ has been split up into its first level administrative division for better visualization of the spread of the epidemic. > 5000 cases per million inhabitants. 2000–5000 cases per million inhabitants. 500–2000 cases per million inhabitants. 200–500 cases per million inhabitants. 50–200 cases per million inhabitants. > 0–50 cases per million inhabitants. No reported cases, no population, or no data available

We used Worldometer to collect national COVID-19 attributable data as of May 2, 2020, which includes “cases per million” and “deaths per million” attributes for the top 183 countries reporting highest case events [7]. We used the same source to collect information on COVID-19 testing data at country level. For BCG coverage, we used WHO-UNICEF estimates of BCG coverage (last update 15 July 2019) [8] (Figs. 1 and 2).

Ethical approval was not required for this report of existing peer reviewed literature.

## Main text

As for data published from the WHO first announcement of the disease to 18th May 2020, when we analyzed the available data on BCG coverage (10, supplement 1), WHO COVID-19 status reports [1] in 183 countries (Fig. 1) and correlated the incidence and mortality patterns from COVID-19 among different countries (Table 1); the following data were noticed. There is a statistically significant negative moderate correlation between BCG coverage and death rates (*P* < 0.001) as well as negative correlation between BCG coverage and incidence rate of all cases as well as serious and critical cases of COVID-19 among the studied countries (*P* = 0.001).

Since the beginning of the pandemic, it was noticed that countries with BCG vaccination program appear to have a lower incidence and death rate from COVID-19 as compared to countries without such a program [9]. Reports showed that morbidity and mortality due to COVID-19 are associated with early adoption or universal coverage of BCG vaccination. They suggested that BCG might show long-lasting protection against SARS-CoV-2 by reduction in the incidence of the respiratory tract infections in children, antiviral effects, and decrease viremia in experimental animals [3, 10]. The vaccine may slow down the spread and progression of symptoms and decrease the number of total cases and deaths per one million [11]. There is a significant difference in the COVID-19-related fatality rates (CFR) between countries with high COVID-19 disease burden and those with BCG revaccination policies [12]. The mortality and reported COVID-19-attributable mortality (COVID-19-related deaths) among BCG-using countries is 5.8 times lower [95% CI 1.8–19.0] than in non-BCG-using countries [13] and in countries with mandated BCG vaccinations compared to countries that terminated BCG vaccination policies before 2000 [14].

However, many reports do not support the BCG hypothesis that all existing claims are based on cross-country correlations [15, 16]. When comparing the same stage in the epidemic, a study does not find any significant difference in COVID-19 severity between countries with or without BCG policies [17]. A recent compares the incidence of COVID-19 infection among vaccinated versus unvaccinated individuals and showed a similar rate of positive test results for SARS-CoV-2 compared with no vaccination [18]. However, this study compared the two groups in terms of laboratory confirmed COVID-19 by RT RNA PCR. But if we assume that BCG vaccinated individuals had more/less asymptomatic infections, the results of this study will be biased as many positive cases will be counted as negative in the analysis. This is called a misclassification error. A future study comparing the two groups with serology testing for anti-SARS-CoV-2 antibodies will be more appropriate.

## Conclusions

BCG vaccine correlates with COVID-19 case fatality rates and probably offers protection against severe/critical cases of SARS-CoV-2. Recommended work through randomized controlled trials to determine how fast a BCG-induced protective immune response against COVID-19 develops is needed to validate its use as immune prophylaxis for more exposed population as healthcare workers.

The findings of the current report should be viewed within the limitation that these data are rapidly changeable; the majority of the studies use publicly available data repositories such as Worldometer to source COVID-19-related data [4]. In the same time, most studies source country-wise BCG-related data using outlets such as BCG World Atlas (http://www.bcgatlas.org/) [8] and other sources, such as reports and datasets published by the World Health Organization, World Bank, and United Nations. Another limitation is that there may be several confounding issues such as limited testing and reporting in many countries. However, these data are derived from 183 countries out of 210 countries reported globally, as of 2:00 a.m. CEST, 4 May 2020. Other limitations include the populations are not stratified by age and comorbidities which may be confounders in case fatality rate from COVID-19. COVID-19 managing protocol is different in different countries. Recent mutations may have occurred in coronavirus, and subtypes caused SARS-CoV-2 disease with different virulence. Lastly, the acquired immunity from BCG lasts for different periods, and there is a strong recommendation to test its validity by tuberculin test or QuantiFeron gamma in COVID-19 patients.

## Data Availability

Available online data.
